# Cardiovascular adverse events in oncology trials: understanding and appreciating the differences between clinical trial data and real-world reports

**DOI:** 10.1186/s40959-022-00139-w

**Published:** 2022-07-19

**Authors:** Michael S. Ewer, Jay Herson

**Affiliations:** 1 Department of Cardiology, Internal Medicine Division, The University of Texas MD Anderson Cancer Center, Houston, TX USA; 2 The Department of Biostatistics, Johns Hopkins Bloomberg School of Public Health, Baltimore, MD USA

**Keywords:** Clinical trial adverse event rates, Post-approval event rates, Reconciliation of event rate discrepancies, Disproportionality analysis, Pharmacoepidemiology

## Abstract

Reports of cardiac adverse events from oncology clinical trials often are at variance with reports derived from clinical observations or data-base reviews. These differences may lead to confusion, as different levels of risks abound in the literature, and the true cardiac risk of using some agents is uncertain. Additionally, such discrepancies may lead to the creation of over-cautious surveillance algorithms. Reasons for these reported differences are complex and often reflect subtleties in the criteria for individual patient evaluation. Both clinical trial data and real-world data have potential flaws that make reconciliation problematic. Importantly, however, both provide crucial information regarding the risk of adverse events. Major factors contribute to these differences including different tools used to diagnose events, and how those tools are interpreted. Additionally, differences in the populations of clinical trial participants and real-world populations play a crucial role. This paper looks at these differences and provides a perspective intended to help clinicians interpret reported variations in event rates derived from highly scrutinized clinical trials and broader real-world data.

## Introduction

Reconciling data regarding adverse events from clinical trials with those subsequently reported from real-world data is challenging and is a timely issue [1]. Pre-approval randomized clinical trials (RCT) for oncologic agents produce data that are collected from subjects chosen with the intent to limit variability; the data are collected under controlled conditions on a limited number of patients who meet the criteria for entry into the trial. RCTs are often undertaken to assess pharmacologic efficacy, but safety considerations may be noted as secondary endpoints. Thus, RCTs may not be powered to detect or quantify unusual but sometimes hugely important adverse events. In considering and comparing cardiovascular events that are reported in oncologic RTCs with those of after-market surveillance, often referred to as *real-world* data (RWD), we may be faced with the dilemma of interpreting differences in the reported incidence of adverse events and in arriving at meaningful assessments of true drug-related risks that are of crucial importance to both clinicians and patients.

Adverse events are reported as either the number of occurrences in a given population or as a statistically significant increase over a known baseline rate for the event. Occurrence rates are reported in settings where the event is considered unlikely to have otherwise occurred, while increased rates over baseline are reported when events are anticipated but have occurred more frequently; the level of significance used commonly in clinical assessments is a *p* value of ≤ 0.05, implying less than a one in twenty chance that the finding is a consequence of random variation. Clinical trial data are our starting point, offering both regulators and clinicians an initial estimate of adverse events; these estimates are imperfect, as they are based on the less diverse trial population and often on a shorter duration of surveillance. In the regulatory setting the RCT may be considered a construct for an approval decision but not the final word on efficacy and safety. Clinical trial data may also be limited by the event thresholds defined in the study protocol. The trial methodology, even when followed carefully, may be over- or underinclusive, as the predictive value of the scrutinized parameter may be less than perfect, and confounding factors may be unavoidable. When the reported data are considered reliable the adverse event and appropriate screening strategies may be incorporated in after-approval clinical monitoring requirements or guidelines. In some instances, surveillance that was suggested or required initially may be questioned or revised, as considerations regarding benefit and cost may arise [2]. Furthermore, the reality that trials of anti-cancer drugs are not, and often cannot be, sufficiently large to identify and quantify some cardiac events that are clinically relevant must be considered.

In the final analysis it is essential to know what clinically relevant adverse events take place with drug exposure. The likelihood of an event, the position of the event on a scale of severity, and the long-term implications of the event at the levels encountered are all crucial parameters, and they are unlikely to be fully defined in RCT data. Notwithstanding this fact, RWD may be problematic for a variety of reasons as well. This paper will explore concerns regarding RCT and RWD data from a clinical and statistical perspective with the goal of furthering and improving our understanding of how these data sets can be interpreted and understood. We will explore why incidence of adverse events might sometimes differ between estimates obtained from RCT and RWD, and how to make best use of both. The discussion is based on cardiac events associated with cancer treatments, but the explanations provided may be relevant to other specialties as well.

## Definitions and methodology

For the purposes of this paper, RCT data refer to published reports from clinical trials that were obtained as part of the trial. Data may have been used to support the application for approval or a component of required post-approval surveillance. Real world data (RWD) refer to sporadic reports of adverse events such as those found in insurance company claims databases, FDA adverse event databases, Medicare claims, and other such databases as well as individually reported events from clinicians. These and similar databases are used routinely in the emerging sciences of pharmacovigilance and pharmacoepidemiology [3, 4]. RCT data are important activities of pharmaceutical companies and regulators to discern adverse events that emerge, often as part of the regulatory process, both pre- and post-approval; RWD comprise information that on the one hand is broader, but on the other may have been derived from less vigorously controlled sources.

Biostatisticians and pharmacoepidemiologists have applied methodology in an attempt to link adverse events with sporadic report data. The chief methodologies are measures of disproportionality. An example is provided by Rothman, Lanes and Sacks, who presented the proportional reporting ratio, i.e., the proportion of reports for a myocardial infarct, related to a drug of interest compared to reports for this event for all other drugs that happen to be in the database under analysis [5]. They also presented a closely related measure, the reporting odds ratio. Both of these measures of disproportionality have been severely criticized for bias due to the drugs that happen to be in the database being mined [6]. In addition, sporadic report analyses are vulnerable to publication bias. If researchers are looking for an increased risk of an agent among sporadic reports, negative findings may be less likely to be submitted or accepted for publication than might be the case where an association was found [7]. Other authors have presented similar methods for using sporadic reports, but pharmacovigilance reports have also been criticized [8, 9].

Many analyses of serious adverse events for anticancer treatments use the reporting odds ratio on data from the FDA Adverse Events Reporting System (FAERS) database that defines serious as: “death, hospitalization, life-threatening, disability, congenital anomaly and/or other serious outcome [10].” A notable example is the analysis of cardio-related adverse events among tyrosine kinase inhibitors (TKI) used for chronic myeloid leukemia [11]. The authors mined the 20 million FAERS reports, 1.3 million related to anticancer drugs; among the many findings were that among anticancer drugs TKIs had a 2.4-fold increased risk of cardiac failure and a 3.8-fold increased risk of ischemic heart disease. For cardiac failure, dasatinib and bosutinib had the highest risk among TKIs. Practitioners may want to exert extra caution when prescribing these agents, however these results deserve some element of skepticism based on the shortcomings of this type of analysis enumerated above.

## Perspective on adverse events

### Taxonomy of adverse events

The actions of pharmaceutical agents are broad, in that multiple pathways are often altered. These agents may precipitate physiologic and pathologic alterations that may go beyond the intended targets of action or may demonstrate unexpected consequences or undesired manifestations of an intended or targeted action. Some such events may be anticipated based on the agent’s pharmacology, while others may arise that had not been foreseen or anticipated. Such events may be the result of augmented expression in a subgroup of patients who are especially sensitive, while others appear sporadically and represent idiosyncratic reactions that are generally unanticipated and unpredictable. All such events fit within a spectrum of expression and consequences that run from the trivial to life-threatening; some may resolve without sequalae, others may cause permanent injury to a tissue or organ, or death.

Toxicities may be thought of as occurring in one of two types: the first will affect the entire population exposed, in that those exposed demonstrate toxicity in a predictable way. Not all will experience the event at the same time nor at the same single or cumulative dose, but the events are predictable statistically and are related to dose, administration, metabolism, and elimination as well as diversity within and among the subjects. Toxicities of this type can be assessed by changes in the mean values of key parameters such as the cardiac ejection fraction, prolongation of corrected QT electrocardiographic interval, or metabolic/biologic markers. The second type of adverse event that is detectable among clinical trial populations are idiosyncratic events; they may be rare, but they can be serious or life-threatening. As they are isolated events, they may not influence the mean value of parameters such as the ejection fraction in a cohort analysis; the mean ejection fraction may not change due to isolated and somewhat random events. These types of events can be assessed by the number of exposed patients meeting specific criteria on a grading scale such as the Common Terminology Criteria for Adverse Events v5.0 (CTCAE) [12]. It is for this second type of toxicity that RWD can provide greater understanding of the risks and help in guiding clinical decisions.

### Clinical trial [RCT] data

As clinical trial protocols for new agents are developed, a wide net is cast to identify both on-target and off-target events and quantify their frequency, severity, and resolution among those exposed. Placebo or comparator arms help to distinguish those events that are related to the agent from those occurring in similar populations who received placebo or alternate regimens. To reduce the number of unrelated events that erroneously might be attributed to the agent of interest, stringent inclusion and exclusion criteria are specified. For example, a clinical trial that looks at oncologic efficacy and has cardiotoxicity as a secondary endpoint might exclude those with baseline LVEF less than 55%, or those with pre-existing cardiac conditions. Such trials may not be sufficiently powered within the limited trial population to identify or quantitate cardiac risks that may come to the attention of clinicians and regulators only when real-world populations are treated, scrutinized, and evaluated.

### Sporadic event data [RWD]

RWD are essential, as they can provide background information about rates of events to compare with toxicities observed in a randomized controlled trial (RCT) and can provide data on patients underrepresented in RCTs. RWD are essential in pharmacovigilance programs to provide an early warning of toxicities too rare to have been observed in RCTs [13–15]. As the practice of medicine evolves and becomes more personalized, this information will be increasingly relevant.

Notwithstanding these considerations, we cannot assume that frequencies of adverse events reported in RWD databases represent the true drug-associated risk for a given agent or combination of agents. Insurance claims are intended to achieve payment for services, not to provide accurate clinical data for research. Claims made to third-party payers are coded and providers’ coders may show variation that result in sub-optimal estimations of actual adverse events. Additionally, events reported in insurance databases may be reported in other databases such as the FDA’s Adverse Event Reporting System as well, providing a potential for double counting. Notwithstanding these considerations, data from sporadic reporting databases result from attention of the reporting clinician which is not likely the case for a routine entry into trial-related database. Additionally, sporadic reporting usually does not provide data on severity and duration of the entered adverse events. Furthermore, events that might occur years after the attributed treatment was ended might be attributed to an agent when the likelihood of causality is low. For the most part such claims may remain in claims databases without the adjudication process required in many clinical trials, and a patient would not be a record in an insurance claims database at all if a claim suggesting an adverse event had not been filed. Epidemiologists refer to this phenomenon informative presence.

Often missing from RWD databases are data on comorbidities, concomitant medication, adherence to medication, baseline data and how cardiovascular measurements are made. Even death might not appear in an insurance claims database because death is not a billable event. These differences are likely to affect the divergence in cardiovascular toxicity rates between RCT and RWD. Indeed, attempts to duplicate RCT results with carefully selected RWD have been challenging [16].

There are limitations to RCT data as well. Due to the stringent eligibility criteria, the patients who enter the RCT are not representative of patients seen in practice and RWD might be more representative. Follow up time is short RCTs compared to RWD, and RCTs are not useful in detecting rare events. For RCTs factors such as adherence and protocol deviations may vary by geographic region [17].

Table 1 presents a comparison of the goal of RCT, admittedly idealized, compared to RWD for various categories of research interest.

**Table 1:** Comparison of randomized clinical trial and real-world data sources

Characteristic	Goal of Randomized Clinical Trial (RCT)	Real World Data (RWD) (Possible confounders noted)
Treatment assignment	Randomization	Agreement to treat between patient and physician (Ideal might be limited due to insurance coverage or other considerations)
Concomitant medications	Specified in protocol	Some patients may be receiving concomitant medications excluded in the RCT protocol. Underreporting of concomitant medications more likely than in RCT
Risk/benefit	Data monitoring committees and cardiac panels scrutinize adverse events and can request further information on adverse-event experiences	Limited to the data in the database or individual event reporting. Insurance data may not include deaths as they are not a billable event
Adverse event reporting	Attempts are made to be complete Events reported in structured format and actively collected as part of the ongoing clinical assessment	Reporting may be less rigid and may be over- or under-inclusive. Diagnosis often not verified, and a tendency not to report well established toxicities may also exist. History may be incomplete
Attribution of a cardiotoxic event to drug of interest	Not always possible to separate drug-induced change from a confounding factor	Not always possible to separate drug-induced change from a confounding factor. Inconsistent reporting of events may compromise the validity of conclusions
Medication and other compliance	Often recorded as per the protocol. Deviations in compliance exist.^17^	Data on drug compliance may not be sought
Clinical endpoints	Pre-specified efficacy and cardiac events adjudicated according to schedule in most trials	Adverse event noted clinically, and conditions of concern may be scrutinized. Endpoint data often not obtained
Confounders	Balance between measured and unmeasured confounders may be handled by randomization	Many unmeasured confounders allow for a more comprehensive overview of adverse events
Inclusion/Exclusion Criteria	Precisely defined in protocol	Patients may have comorbidities or take concomitant medications that are not allowed in the RCT and are not reported in database
Baseline variables	Attempts made to limit variables by inclusion and exclusion criteria	Often baseline variables not adequately measured
Cardiovascular data	Consistently measured using standardized techniques at defined intervals	Technique and timing may vary

Adverse event incidence in RCTs can be further refined through meta-analysis as was demonstrated in a recent paper on bevacizumab on more than 20,000 patients across 22 RCTs [18, 19]. Specifically, the authors pooled the odds ratios of arterial and venous adverse events and found a statistically significant 37% increased risk on bevacizumab treatment. Meta – analysis provides a test of heterogeneity across trials. The authors found no evidence of heterogeneity and, thus, pooling the trials was permissible. In reviewing reports of meta-analyses in the literature physicians should be aware of certain biases and pitfalls in the analyses. Meta-analyses may be performed on aggregate patient data (APD) or individual patient data (IPD). APD analysis is performed by extracting data from published clinical trials. Indeed, some trials might be deleted from analysis because the needed data, such as LVEFs, were not in the publication or not clearly defined. IPD analyses pool actual patient data from similar trials and may overcome potential publication bias. The Early Breast Cancer Trialists Collaborative Group, established in 1983, performs IPD analysis pooling data from member institutions [20].

Other issues in meta-analyses arise if the active control treatments and/or combination therapies were not the same across trials being analyzed. Similarly, eligibility requirements may have varied over time. Technologies for diagnosing cardiovascular adverse events may have changed as well. There might be a difference in the timing and frequency of follow up visits where adverse events are assessed. Patients on trials in the past might have had longer exposure to experimental treatment than in more recent trials because in the past there were fewer or no alternative treatments available. Finally, meta-analyses are also classified as fixed effect vs. random effect analyses. The latter introduces extra sources of variation among trials which is useful to absorb the types of variation mentioned above and would be considered preferable to fixed effects analysis.

In evaluating drug safety, a migration from clinical trial data to real-world data may present huge concerns. We provide an illustrative example.

### Osimertinib, an example of low toxicity in both RCT and RWD, but with on-going concerns regarding late toxicity

Osimertinib is a third-generation, irreversible, oral epidermal growth factor receptor tyrosine kinase inhibitor. Cardiac events were sought during 2 clinical trials and in post-approval populations. The RCT event rate for declines in ejection fraction, defined as a drop of ≥ 10 percentage points to a value of < 50% for the pooled population was 3.9% and most events were considered reversible. The authors concluded that “these data do not suggest a causal relationship between osimertinib and cardiac failure [21].” However, individual case reports of cardiac failure have emerged, suggesting causality, especially in an older population with advanced malignancy [22]. Reconciliation of these data are challenging: a retrospective report noted a cancer therapy-related cardiac dysfunction incidence of 4.4 percent and concluded that osimertinib was associated with a risk of dose-independent and reversible cancer treatment-related cardiac dysfunction; surveillance artifact was not considered in this analysis [23]. The data are remarkably consistent and differences are understandable when methodology, population variance, and confounding factors are considered. Nevertheless, conclusions regarding true cardiac risk remain at variance suggesting that better criteria, more consistent definitions, universally accepted measuring tools, and new analytical strategies are needed to put such controversies to rest and allow a more objective assessment of risk for cancer patients. At present the RCT and RWD present the oncologist with data to integrate with their knowledge and judgment for risk–benefit decisions that best serve their patients.

## The natural evolution of data from clinical trial to real-world

We start with clinical trial data recognizing that we have a selected group that fundamentally may be different from the cohorts that are ultimately treated in the post-approval period. Such data provide an initial estimate that, in the final analysis, is an imperfect tool for the ultimate definition of future risk. As we accumulate real-world data and gradually expand our knowledge base using what is reported, we must be ever cognizant that in real-world databases, biases may be introduced, confounding factors may result in over- under-inclusion, and criteria for triggering an event may be problematic. Ideally RCT should, over time, yield to broader RWD data, with the caveat that RWD must evolve to be reliable, reproducible, and not burdened with confounding variables and false-positive or false-negative assessment data [24]. Additionally, we must recognize that often clinicians go down a path that has been established for surveillance and reporting; that path may be difficult to change over time, even when data suggest that a modification is appropriate. As new data become available, we may discover that our established surveillance algorithms may be either insufficient or excessive, and that suggestions published in the form of expert opinions or guidelines need to be reevaluated and updated to optimize their intended benefit for our patients.

## Discussion

We have given some reason for caution in the use of both RCT data and RWD to evaluate cardiovascular adverse events for drugs. As clinicians, we must appreciate the need for RWD as it may provide an early warning of adverse events that could not have been observed in the limited premarket populations. When a drug enters its post approval era, we cannot totally rely on premarket data as we consider if the drug is sufficiently safe for all demographic groups, especially those in populations who were not included in the clinical trials.

Solutions to these enigmas are foreseeable but will take time to integrate into standard clinical practice. They are likely to involve record linkage, artificial intelligence, and machine learning [25]. They will also require the flexibility to re-think our approach to patient evaluation, surveillance, and reporting so that the data can be interpreted broadly yet applied to subgroups rationally. Regarding cardiac sequalae of cancer or cancer treatment, the disciplines should work together to integrate databases for broader comparison. New methodology would expand the now routine use of electronic health records (EHR) in medical practice to allow broader reporting. Record linkage, for example, could locate a spontaneous report in an insurance claims database and link that record to this patient’s EHR where needed data on concomitant medicine, comorbidities, serial electrocardiograms, and other data could be then included in the analysis [26, 27]. Researchers interested in searching for cardiovascular adverse events among patients being treated with a specific agent might start by searching claims for this adverse event in a claims database but could also link each patient claim entry to the patient’s EHR or cancer registry entry in order to retrieve medical history, comorbidity and concomitant medications. A more complete analysis could then apply artificial intelligence and machine learning to reach conclusions with far greater accuracy than is possible using present methods. Project One Source is developing computer methodology for the continuous efficient transfer of structured data from HER to a common research format [1]. We are learning the limitations and challenges of ad-hoc cancer research databases [28]. When taken together these strategies should offer a big step forward in the understanding of cardiovascular and other risks related to pharmacologic agents. The Sentinel Initiative, a collaboration of FDA and many large health insurance companies, EHR records, patient reports which pool their data provide a rich data resource for development of new analytic methods and risk assessment, is certainly a part of the future of cardiovascular safety, and is an important step in the direction of data integration [29]. It is anticipated that in the near future the sources of RWD will evolve and become centralized to provide extensive data on adverse events, perhaps achieving overviews that more resemble their RCT counterparts, yet be more comprehensive and more accurate in their assessments. Until we have more sophisticated integration of healthcare databases, we should neither try to treat RWD as RCT data, nor vice versa; they represent independent sources of adverse event experience to be interpreted within the confines of their individual strengths and weaknesses.

## Data Availability

Not applicable.
